# Annual Commencement of Niagara University Medical College

**Published:** 1897-06

**Authors:** 


A Monthly Review of Medicine and Surgery.

*EDITORS:*

THOMAS LOTHROP, M. D. - - WM. WARREN POTTER, M. D.

All communications, whether of a literary or business nature, should be addressed to the managing editor: 284 Franklin Street, Buffalo, N. Y.

Vol. XXXVI. JUNE, 1897. No. 11.

### ANNUAL COMMENCEMENT OF NIAGARA UNIVERSITY MEDICAL COLLEGE.

THE twelfth annual convocation of the medical department of Niagara University was held on Wednesday, May 12, 1897. Following our usual custom we give a detailed account of the exercises, which each year becomes a part of the history of medicine in Buffalo. The value of such a record in the Journal will be more and more appreciated by both faculty and alumni as years advance.

### ALUMNI ASSOCIATION.

The annual meeting of the alumni association was held at the college building, on Ellicott street, and began at 10.30 o'clock A. M. with an address of welcome by Dr. John Cronyn, president of the medical faculty. In his address Dr. Cronyn said that the function of an alumni association was to stimulate the interest of the graduates in their alma mater. A plan of work, he said, was necessary to the success of an alumni association and he suggested that the association prepare today for the work of its next meeting. Dr. Cronyn talked briefly about the work of the graduates after they have begun practice.

In the absence of the president, Dr. Jacob S. Peterson, of New York, the president's annual address was omitted. The first vice-president, Dr. Frank W. Maloney, of Rochester, presided and during the business session the following-named officers were elected to serve during the ensuing year: president, Dr. Frank W. Maloney, of Rochester; first vice-president, Dr. Frederick A. Hayes, of Buffalo; second vice-president, Dr. William G. Taylor, of Buffalo; secretary, Dr. J. G. Ernest, of Buffalo; treasurer, Dr.

Frederick M. Boyle, of Buffalo ; executive committee, Drs. Joseph J. Finerty, J. Henry Dowd and Henry C. Buswell, all of Buffalo.

The afternoon session opened at 3 o'clock, when the following program was disposed of : Among the paupers, Alfred W. Bayliss, M. D. ; The importance of insurance medicine, Frank W. Maloney, M. D. ; The Balanos or distal inch, B. H. Daggett, M. D. ; Chronic specific urethritis, J. Henry Dowd, M. D.

Great credit is due to the executive committee, composed of Dr. J. J. Finerty, chairman, Drs. Frederick A. Hayes and J. G. Ernest, for the successful alumni meeting and for the complete arrangements for the banquet at the Genesee in the evening.

### GRADUATING EXERCISES.

The graduating exercises were held at the Star Theatre on the evening of commencement day, beginning at 8 o'clock. The theatre was crowded at an early hour and soon after the time appointed for the beginning of the exercises members of the faculty and the speakers of the evening seated themselves upon the stage. When the curtain went up, the graduates took their places in the first row.

Dr. John Cronyn officiated as master of ceremonies.

The valedictory address was delivered by Dr. John A. Miller, professor of medical chemistry and toxicology at the university.

It was announced that Right Rev. James E. Quigley, D. D., chancellor of the university, would confer the degrees, but he was unable to attend and appointed Rev. Father McHale, president of Niagara University, to act in his place.

The names of the candidates were then called by Dr. H. A. Wood and the degrees were conferred. The new graduates are Alexander McNiel Blair, Pierce John Candee, Charles Joseph Mengis, William Keating O. Callaghan, Walter Landor Savage, Henry Edward Stadlinger, Buffalo ; Leonard Ezra Curtice, Springwater, N. Y. ; Arkadius Prokofieff Jukowsky, Russia ; William John O'Donnell, Ellicottville, N. Y. ; Mary O'Malley, Barker's, N. Y.

The manner of conferring the degrees is by the ancient rite of hooding, which is widely used in English universities. Each candidate is introduced by a graduate to the chancellor in a few set Latin words. The candidate then kneels and the chancellor takes him by the hand and replies to the introduction. While the candidate is kneeling, another graduate, acting as beadle, hoods him.

After this ceremony the class took part in the affirmation of the pledge to promote morality and philanthropy, as well as to continue zealous in their profession.

The annual address was delivered by Rev. Frank S. Fitch, pastor of the First Congregational Church. He made a profound impression upon the class as well as on the audience which overflowed the capacity of the house. He congratulated the class members, not only upon their advent into the field of medicine, but into that of philanthropy also. He believed, notwithstanding the current opinion, that physicians did more good for less financial remuneration than any other body, excepting, perhaps, the ministry. The address was replete with sound advice and noble sentiment.

Rev. Father McHale, of Niagara Falls, followed Mr. Fitch with a short and timely address.

Drs. Callaghan, O'Donnell and Stadlinger led this year's graduating class. Dr. Callaghan secured the highest marks in his class. In four subjects he was marked 100 and the rest of his standing was high. He is very popular at the university and received the hearty congratulations of his many friends.

Dr. O'Donnell, one of the surgeons at the Emergency Hospital, stood first in his class. He also received 100 in four subjects. He is the poet and orator of the university. Dr. Stadlinger was applauded loudly when his name was called and he had received his degree. He is a diligent student and made a splendid record.

Dr. Mary O'Malley, distinguished as the only woman graduate, deserves personal mention as a close student.

### THE BANQUET.

At the close of the commencement exercises, the alumni and faculty adjourned to the Genesee, where they enjoyed the alumni banquet.

The visiting graduates included many from nearby and distant cities. Among the guests were Dr. N. G. Richmond, of Fredonia, and Dr. F. W. Ross, of Elmira.

Covers were laid for about seventy. At the conclusion of the material feast, which was near midnight, Dr. Frank W. Maloney, of Rochester, first vice-president, acting as toastmaster introduced the speakers, each in a few well-chosen words. The following toasts were responded to: The University, Rev. L. A. Grace; the Faculty, Prof. Eugene A. Smith, M. D.; Legal Medicine, Mr. E. R.

O'Malley; The State Medical Examining Board, Dr. William Warren Potter; The Medical Profession, Dr. L. A. Weigel, Rochester; The Clergy, Rev. Frank S. Fitch; The Press, Mr. B. Frank Coe; University Board of Examiners, Dr. Frank W. Ross, Elmira; The Graduating Class of '97, Dr. Pierce Candee.

It was long after midnight when adjournment came and there were many expressions of approval on all hands at the eminently successful closing of the thirteenth academic year, the pleasant conduct of the convocation ceremonies, and of the delightful banquet that crowned the whole in fitting manner.

